# Mechanosensory Neuron Aging: Differential Trajectories with Lifespan-Extending Alaskan Berry and Fungal Treatments in *Caenorhabditis elegans*

**DOI:** 10.3389/fnagi.2016.00173

**Published:** 2016-07-18

**Authors:** Courtney Scerbak, Elena M. Vayndorf, Alicia Hernandez, Colin McGill, Barbara E. Taylor

**Affiliations:** ^1^Institute of Arctic Biology, University of Alaska FairbanksFairbanks, AK, USA; ^2^Department of Biology and Wildlife, University of Alaska FairbanksFairbanks, AK, USA; ^3^Department of Biology, Earlham CollegeRichmond, IN, USA; ^4^Chemistry Department, University of Alaska AnchorageAnchorage, AK, USA

**Keywords:** mechanosensory neuron, *C. elegans* neuron aging, neuron morphology, nutrition, blueberry, lowbush cranberry, chaga, crowberry

## Abstract

Many nutritional interventions that increase lifespan are also proposed to postpone age-related declines in motor and cognitive function. Potential sources of anti-aging compounds are the plants and fungi that have adapted to extreme environments. We studied the effects of four commonly consumed and culturally relevant Interior Alaska berry and fungus species (bog blueberry, lowbush cranberry, crowberry, and chaga) on the decline in overall health and neuron function and changes in touch receptor neuron morphology associated with aging. We observed increased wild-type *Caenorhabditis elegans* lifespan and improved markers of healthspan upon treatment with Alaskan blueberry, lowbush cranberry, and chaga extracts. Interestingly, although all three treatments increased lifespan, they differentially affected the development of aberrant morphologies in touch receptor neurons. Blueberry treatments decreased anterior mechanosensory neuron (ALM) aberrations (i.e., extended outgrowths and abnormal cell bodies) while lowbush cranberry treatment increased posterior mechanosensory neuron (PLM) aberrations, namely process branching. Chaga treatment both decreased ALM aberrations (i.e., extended outgrowths) and increased PLM aberrations (i.e., process branching and loops). These results support the large body of knowledge positing that there are multiple cellular strategies and mechanisms for promoting health with age. Importantly, these results also demonstrate that although an accumulation of abnormal neuron morphologies is associated with aging and decreased health, not all of these morphologies are detrimental to neuronal and organismal health.

## Introduction

Aging is a ubiquitous process affecting the health of increasing numbers of aged individuals throughout the world. Gradual declines in many physiological functions accompany increased chronological age and are associated with increased mortality. Thus, development of strategies to improve tissue, system, and organismal function during aging is an increasing public health priority. Alaskan traditional ecological knowledge holds that a diverse array of local berries, plants, and fungi benefit health and wellness. While plant matter consists of a low proportion of total energy intake in traditional Alaska Native diets (<3% compared to 90% from fish and game meat and fat; Bersamin et al., 2007), plants and fungi historically were and currently are highly valued by Alaska Native traditional healers (Loring and Gerlach, 2009). Various berries, plant greens, and fungi are consumed as part of a standard subsistence diet and used by traditional healers and contemporary herbalists to combat health problems ranging from stomach and muscle pain to bleeding and snow blindness. An increasing number of studies show that transitioning away from Alaska Native traditional diets and lifestyles is associated with increased incidence of age-associated disorders, including cardiovascular disease (Ebbesson et al., 2005; Loring and Gerlach, 2009). Importantly, cultures throughout the world value plants and fungi related to Alaskan species in traditional foods and medicines (Iriti et al., 2010; Kim and Song, 2014).

The nematode *Caenorhabditis elegans* has homologous neuronal features to humans that are vital for nervous system function, which makes these animals a powerful model for studying neuronal aging *in vivo*. Neither healthy brain aging in humans nor neuron aging in *C. elegans* are characterized by cell death (Herndon et al., 2002; Yankner et al., 2008). Instead, age-related cognitive and functional decline in the human brain is associated with neuroanatomical changes, such as decreased white matter (i.e., myelinated neuron axons, glial cells), altered dendritic branching, and decreased synaptic density (Yankner et al., 2008), as well as decreased coordination and altered localization of neuron/neural network activation (Bishop et al., 2010). Recently, certain classes of *C. elegans* neurons, including mechanosensory or touch receptor, neurons have been shown to change morphologically with age (Pan et al., 2011; Tank et al., 2011; Toth et al., 2012). Mechanosensory neurons are central to an organism’s ability to sense and respond to its environment. These neurons, including the anterior lateral (ALM) and posterior lateral mechanosensory (PLM) neurons, develop novel outgrowths from the soma and processes, and deteriorated synapses with age, which can be observed with fluorescently-tagged (GFP) genes and electron microscopy (Pan et al., 2011; Tank et al., 2011; Toth et al., 2012). Assessing the function and phenotypes of mechanosensory neurons within an individual, provides a powerful model for exploring mechanisms of neuronal aging and neurological effects of medicinal Alaskan berries and fungus.

Modifying diet, specifically consuming fruits, vegetables, nuts, and specific spices (e.g., turmeric, which contains curcumin), is proposed to be a practical method to lower age-related cognitive decline (Joseph et al., 2009). Alaskan plant and fungus species have adapted to extreme environments, in part by producing a wide variety of secondary metabolites, bioactive molecules not required for plant growth and development (Wink, 2003; Elks et al., 2013). Various plant and fungal extracts composed of secondary metabolites interact with specific molecular targets to improve health and to slow the progression of aging and age-related disease damage (Liu et al., 2005; Wiegant et al., 2008; Youl et al., 2010). For example, a large body of research describes the anti-inflammatory and antioxidant activity of polyphenolic compounds, such as anthocyanins (Zafra-Stone et al., 2007). Berries in particular have been heavily studied for their impact on brain signaling and neurodegeneration (Spencer, 2008; Miller and Shukitt-Hale, 2012; Shukitt-Hale, 2012).

Alaskan berry species consistently contain higher levels and activity of antioxidant and anti-inflammatory compounds (e.g., phenolic, flavonoid, and anthocyanin compounds) than other commercially grown, temperate species (Kalt et al., 2001; Dinstel et al., 2013; Grace et al., 2014). The selected berries in this study, bog blueberry (*Vaccinium uliginosum*, also known as bog bilberry), lowbush cranberry (*Vaccinium vitis-idaea*, also known as lingonberry), and crowberry (*Empetrum nigrum*, also known as blackberry or mossberry), are found throughout Alaska and the circumpolar north in bogs, woodlands, and spruce stands. Non-Alaskan species of lowbush blueberries (*Vaccinium angustifolium)* prevent and reverse object-recognition memory loss in aging rats (Joseph et al., 1999) and improve memory in both children (Whyte et al., 2015) and older humans (Krikorian et al., 2010). Blueberry polyphenols (*V. angustifolium)* also extend wild-type *Caenorhabditis elegans* lifespan (Wilson et al., 2006). The American cranberry (*Vaccinium macrocarpon)* extends *C. elegans* lifespan in a dose-dependent manner (Guha et al., 2012). Crowberry contains higher total anthocyanins than other well-studied berries, such as blueberries, raspberries, and cranberries (Ogawa et al., 2008), but little is known about its biological actions. Thus, we set out to investigate the benefits of consuming Alaskan berries.

Chaga (*Inonotus obliquus*, also known as cinder conk) is a parasitic fungus found on birch trees; it has a long history of a wide variety of medicinal uses in Asia and Eastern Europe, as well as in Alaska. Chaga extracts exhibit high antioxidant activity (Cui et al., 2005) and protect rat neuronal cells against oxidative stress (Giridharan et al., 2011). Korean traditional knowledge heralds chaga for its anti-cancer effects (Kim and Song, 2014) and ergosterol peroxide extracted from chaga was recently shown to inhibit cell growth by promoting apoptosis in a colorectal cancer cell model (Kang et al., 2015). Taken together, consumption of plants and fungi, especially those biochemically adapted to life in the Arctic, is a promising approach to combating age-related declines in function and development of disease.

The current study aims to describe the influence of Alaskan medicinal berry and fungal treatments on the aging process. We tested the hypothesis that treatments with blueberry, lowbush cranberry, crowberry, and chaga increase wild-type *C. elegans* lifespan and alter touch receptor neuron aging, potentially through different cellular mechanisms. Specifically, we tested the impact of specific treatments on lifespan and healthspan (i.e., motility, touch response, endogenous ROS). By examining the six *C. elegans* touch receptor neurons throughout adulthood using fluorescence microscopy, we also described the effects of the treatments on age-related morphological changes. Studying the impact of these culturally relevant foods on aging not only provides further support for their ethnomedicinal use, but also gives unique insights into the mechanisms of whole organism and neuronal aging.

## Materials and Methods

### *C. elegans* Strains and Maintenance

The following strains were used in this study: N2 (Bristol), ZB154 (zdIs5 [P*mec*-4GFP*; lin-15*(+)]), and TJ375 (*gpIs1*[hsp-16.2::GFP]). We used standard methods to maintain and manipulate *C. elegans* populations (Brenner, 1974). Stock populations were cultured at room temperature (about 22°C) on Nematode Growth Media agar plates (1 L NGM: 2.5 g peptone, 17 g agar, 3 g NaCl, 975 mL double distilled water, 1 mL 5 mg/mL cholesterol, 1 mL 1 M CaCl_2_, 1 mL 1 M MgSO_4_, 25 mL 1 M KHPO_4_, and 0.5 mL 100 mg/mL streptomycin) seeded with live bacteria (*E. coli* strain OP50-1 cultured in Luria Broth) that were allowed to form a lawn for 48 h at room temperature and then stored at 4°C until use.

### Berry and Fungus Extract Preparation

We collected wild specimens of the selected species of natural berries and fungus from Fairbanks, Alaska in late summer and early fall 2012. A local Alaskan mycologist verified the identity of chaga (*Inonotus obliquus)*. All samples were stored whole and fresh frozen at -80°C until extraction. We prepared crude berry extracts by blending and homogenizing ∼200 g frozen berries with 500 mL chilled 80% aqueous acetone (∼4°C) for 10 min and removing the acetone with a rotary evaporator (∼120 min). Specifically, 203.7 g of blueberries, 204.1 g of crowberries, and 200.6 g of lowbush cranberries were extracted to final volumes of 334, 300, and 260 mL, respectively. We prepared crude chaga extract by steeping 19.25 g of ground inner, deep orange core, of the fungus in 50 mL of ∼80°C water and filtering out leftover particulates, to mimic common consumption of the fungus as a tea-like infusion. We determined the stock concentrations of extracts using a ratio of the starting mass of berry or fungus to the final volume of extract. Extracts were then aliquoted and stored at -80°C. Freeze thaw cycles were limited to one (thawed from -80°C then aliquoted and frozen at -20°C until use) to ensure maximal preservation of bioactive components (Lohachoompol et al., 2004). Because seasonal weather patterns and location (e.g., bog versus mountain) have been shown to influence the chemical makeup of various botanicals (Howell et al., 2001), all experiments were conducted using extracts prepared from the same extraction batch to eliminate the potential for differences in the chemical properties of the extracts.

### Biochemical Quantification of Extracts

We quantified total phenolic content of each of the Alaskan berry and fungus extracts using a Folin–Ciocalteu assay with a gallic acid standard curve adapted for 96-well plate analysis (Herald et al., 2012). We also measured flavonoid content with a NaNO_2_/Al/NaOH assay with a catechin standard curve (Herald et al., 2012). The gallic acid and catechin standard curves used for comparison had *R*^2^ values of 0.98 and 0.96, respectively. We quantified anthocyanin content by pH-differential assay with cyanidin-3-glucoside equivalent (Song et al., 2013). Each measurement included at least three technical replicates.

### Berry and Fungus Treatment Administration

To administer Alaskan berry and fungus treatments, we created treatment agar plates by mixing the appropriate concentration of extract into NGM after the agar cooled but prior to it solidifying. Control plates consisted of NGM plates with no extract. We seeded all plates with live OP50-1 *E. coli* cultured from single bacterial colonies in Miller Luria-Bertani (LB) Broth containing 50 μg/mL streptomycin. During all experiments, we ensured that the experimenter was blinded to the treatment groups. In the following described experiments (unless otherwise mentioned), synchronous populations of the appropriate *C. elegans* strains were created on standard, untreated NGM agar plates using the egg lay method (i.e., allow 30 gravid adults to lay eggs for 4 h, then removing the adults), fed standard live OP50-1 *E. coli*, and cultured at 25°C. We transferred experimental populations by hand to treatment plates at the late L4 larval stage, just before adulthood (48 h at 25°C after egg lay), allowing the animals to ingest the extract after normal larval development.

### Lifespan Analysis

Following the production of age-synchronous L4 populations via egg lay, we transferred two replicates of 25 animals onto plates for each berry and fungus treatment. We continued to transfer animals at least every other day of adulthood to fresh, seeded agar plates with the appropriate treatment. After all treatment groups stopped producing eggs, we continued to determine survival at least every other day by visual observation or gentle prodding with a platinum wire. At any point during the experiment, animals with protruding intestines, internal live young, or that crawled off the plates were censored. Survival experiments always included an untreated control in parallel with berry and fungus treatments and were repeated at least three times. For lifespan experiments using UV irradiated bacteria, berry and fungus treatment administration and agar plate preparation were performed in the same manner as all other experiments with the additional step of exposing prepared OP50-1 lawns to 9999 J/m^2^ 254 nm UV light using a UV Stratalinker 1800 and testing for survival before use. We used Kaplan–Meier log-rank survival statistics to analyze differences in mean survival between treatment groups. For this and all other statistical analyses described, we used the statistical software SPSS (version 20) and considered a *p*-value of less than 0.05 statistically significant.

### Motility Measurement

We divided aging individuals’ motility into three classes: A, B, and C, following the methods of Herndon et al. (2002). Class A individuals moved spontaneously in a normal, sinusoidal pattern. Class B individuals moved in markedly non-sinusoidal movements and may have required prodding to encourage movement. Class C individuals moved their head and/or tail in response to prodding, but were unable to move across the agar. Treatment effects were compared to their age-matched no treatment control using an ordinal logistic statistics model.

### Reactive Oxygen Species Quantification

To begin treatment on large age-matched populations, we washed late L4 populations (48 h after bleaching at 25°C) onto treatment plates with M9 (autoclaved 3 g KH_2_PO_4_, 5 g NaCl, 6 g Na_2_HPO_4_^∗^7H_2_O and sterile 1 mL 1 M MgSO_4_ in 1 L H_2_O). On test day (48 h after treatment, day 3 of adulthood), we isolated 300–500 adult worms from each treatment group by washing populations through a 50 μm filter with M9. We then quantified reactive oxygen species (ROS) using H_2_DCF-DA (Halliwell and Whiteman, 2004). To do this, we: transferred the live adults into labeled 2 mL tubes using 1.5 mL M9 buffer; allowed them to settle by gravity; removed the supernatant such that 500 μL of M9 was left in the tube; and pipetted three aliquots of 50 μL of the well-mixed M9-worm solution to the appropriate 96-well plate well. Immediately before loading the plate into the Biotek Synergy^TM^ HT Multi-Mode Microplate Reader, we added 50 μL of 100 mM 2′,7′-Dichlorofluorescin diacetate (DCF-DA, Sigma) in M9 to each well. We recorded basal fluorescence (excitation 485 nm, emission 520 nm), stored the plate in the dark at room temperature on a shaker for 1 h, and recorded final fluorescence. We normalized fluorescence results to basal worm and basal DCF-DA fluorescence (50 μM DCF-DA in M9 buffer). Results were also normalized to total protein in each tube (using the leftover 200 μL of M9-worm solution in the 2 mL tube, described above) using the Pierce BCA Protein Assay on the supernatant following worm lysis (20 μL 1 M NaOH to each tube incubated for 25 min at 70°C). This ROS quantification protocol was performed three times and was adapted from other published protocols (Braeckman et al., 2002; Schulz et al., 2006).

### Fecundity Measurement

To determine whether fecundity, specifically the number of viable progeny produced, was affected by treatments, we performed progeny count assays. We individually plated and transferred age-matched adults each day to fresh plates until the end of their reproductive phase or death. We allowed embryos left behind by the adults each day to develop for 48 h at 25°C and then manually counted the number of progeny produced by each individual on each day. We compared the total number of progeny produced per treatment and control using one-way ANOVA and Tukey *post hoc* comparisons. We tested for treatment effects on each day of adulthood using a Poisson log linear statistical model.

### Mechanosensory Neuron Aging Assay

We performed neuron aging assays (i.e., soft touch response and mechanosensory neuron imaging) using the *C. elegans* strain *zdIs5*, which expresses green fluorescent protein (GFP) in each of the six touch receptor neurons (**Figure 3A**). On days 5, 7, 9, and 11 of adulthood, we randomly selected individuals from a synchronous population, tested for touch sensitivity, and imaged the number and type of specific neuronal aberrations seen in the six fluorescently labeled neurons. We measured soft touch sensitivity and observed mechanosensory neuron aberrations as established by Toth et al. (2012), by counting the number of positive responses an individual had to five alternating touches at each the anterior and posterior ends (10 total touches). To image the fluorescently labeled neurons, we then mounted that individual on a labeled coverslip with 36% Pluronic solution and quantified neuron morphology with an Olympus FSX100 inverted fluorescent microscope at 20× magnification (for an overall magnification of 200×). Neuron morphologies observed included those previously described (Pan et al., 2011; Tank et al., 2011; Toth et al., 2012; Scerbak et al., 2014), such as various lengths of outgrowths from the soma, branches from the process, abnormally shaped soma, punctae on the process, and soma in the wrong location. This assay of mechanosensory neuron form and function was repeated at least three times for each selected Alaskan berry and fungus lifespan-extending treatment and each replicate contained its own distinct untreated control group. We performed Poisson log linear (for count data, i.e., number of outgrowths) or logistic regression (for bimodal data, i.e., presence of abnormal cell soma) statistical models to test for treatment and age effects on touch sensitivity and neuron morphologies. Pairwise comparisons with *p* ≤ 0.05 were considered significant.

### *hsp16-2*::GFP Gene Expression Assay

*hsp-16.2*::GFP reporter gene expression assays were performed on age-matched day 3 adults cultured on appropriate berry and fungus treatment plates at 20°C using 8× magnification on an Olympus FSX100 inverted fluorescent microscope (for an overall magnification of 80×) and constant exposure. Positive control animals were heat shocked at 37°C for 90 min, 20 h before imaging (Rea et al., 2005). To detect treatment effects, we used a one-way ANOVA with Tukey *post hoc* statistical tests.

## Results

### Standardization of Alaskan Plant and Fungus Extracts

Polyphenolic compounds, including flavonoids and anthocyanins, exhibit potent antioxidant and anti-inflammatory properties (Joseph et al., 2014). We quantified three measures of phenolic content to compare the extracts used in this study with others shown to have bioactive properties and other Alaskan berry extracts shown to contain high phenolic content. Total phenolic content (Folin–Ciocalteu assay with gallic acid equivalent), flavonoid content (NaNO_2_/Al/NaOH assay with catechin equivalent), and anthocyanin content (pH-differential with cyanidin-3-glucoside) of each extract were measured using established methods (**Table 1**) (Herald et al., 2012; Song et al., 2013). The *relative* abundance of total phenolics and anthocyanins of blueberry compared to lowbush cranberry levels was the same as previously reported for Alaskan blueberry and lowbush cranberry (Grace et al., 2014); total phenolic content was 443% higher in lowbush cranberry than blueberry while anthocyanin content was 137% higher in blueberry than cranberry (**Table 1**). The crowberry extract contained the highest levels of total phenolics and anthocyanins of all the extracts. In contrast, the chaga extract contained the lowest levels of total phenolics, flavonoids, and anthocyanins of all the extracts.

**Table 1:** Biochemical quantification of crude extracts.

	Total phenolic content (mg GAE g^-1^ FW)	Flavonoid content (mg CAE 100 g^-1^ FW)	Anthocyanin content (mg C3G L^-1^ FW)
Blueberry	190.4 ± 4.0	202.3 ± 1.4	217.4 ± 5.3
Lowbush cranberry	219.6 ± 6.4	896.2 ± 8.6	158.4 ± 5.3
Chaga	74.9 ± 3.3	125.9 ± 3.1	5.5 ± 2.6
Crowberry	365.3 ± 9.2	453.2 ± 30.1	713.9 ± 45.2

*Total phenolic content (quantified by Folin–Ciocalteu assay with gallic acid equivalent), flavonoid content (quantified by NaNO_2_/Al/NaOH assay with catechin equivalent), and anthocyanin content (quantified by pH-differential with cyanidin-3-glucoside) ± standard error of the mean for each berry and fungus extract is reported*.

### Alaskan Berry and Fungus Treatments Extend *C. elegans* Lifespan

**FIGURE 1:**
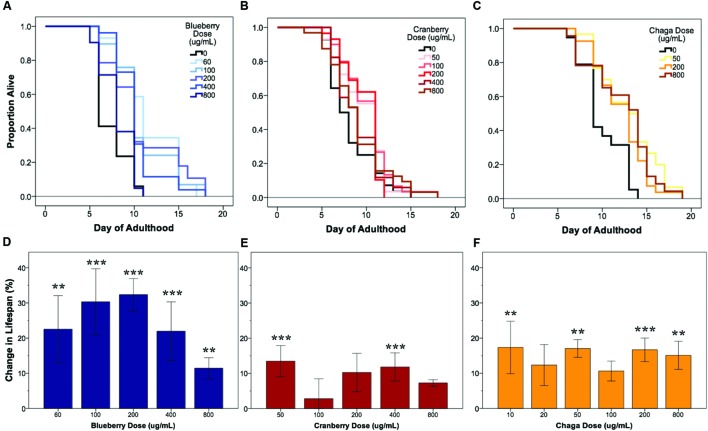
**Alaskan berry and fungus treatments extend wild-type *C. elegans* lifespan**. Representative survival curves for each berry and fungus treatment **(A–C)** and induced percent change in lifespan relative to control for all replicates **(D–F)** are shown (*N* = 50 per treatment group). Asterisks denote significant lifespan extension (*p* < 0.05; Kaplan–Meier log-rank test) from control in 75% or more (^∗∗∗^) and 60% or more (^∗∗^) of 4–6 independent trials. Bars represent mean ± standard error of the mean between all replicates of a treatment dose.

Under our laboratory culture conditions (i.e., 25°C, solid media, live OP50-1 *E. coli* food source), *C. elegans* untreated control mean lifespan was 10.2 ± 0.4 days. Three of the four tested Alaskan berry and fungus treatments resulted in wild-type *C. elegans* lifespan extension: blueberry, lowbush cranberry, and chaga treatments extended lifespan at varying doses and to different extents when compared to untreated control populations (**Figure 1** and **Table 2**). Blueberry treatment elicited the highest increase in lifespan of all tested Alaskan berries and fungus; an average of 30% and up to 47% (200 μg/mL treatment) when compared to untreated control. Blueberry was also the only treatment for which an entire range of doses tested (60–400 μg/mL) resulted in lifespan extension (**Figure 1D**). In a representative trial, mean lifespan significantly increased over the no treatment control to 12.4 ± 0.6, 11.2 ± 0.6, 12.4 ± 0.7, and 11.2 ± 0.6 days upon treatment with 60, 100, 200, and 400 μg/mL Alaskan blueberry extract, respectively (*p* < 0.05, Kaplan–Meier log-rank test). Treatment with 800 μg/mL blueberry did not consistently, statistically increase lifespan.

**Table 2:** Alaskan berry and fungus treatments extend wild-type *C. elegans* lifespan.

Treatment	*N*	Mean lifespan ± SEM	Percent of control	*p*-value	Highest increase observed	Lowest increase observed
**Blueberry dose (μg/mL)**	
0	60	9.48 ± 0.31		
60	66	12.4 ± 0.56	156%	<0.0001	128%	107%
100	60	11.2 ± 0.59	147%	<0.0001	144%	111%
200	51	12.4 ± 0.73	146%	0.001	147%	120%
400	58	11.2 ± 0.58	139%	<0.0001	139%	120%
800	60	9.31 ± 0.28	109%	0.341	117%	109% (n.s.)
**Lowbush cranberry dose (μg/mL)**	
0	50	11.3 ± 0.58		
50	50	13.9 ± 0.91	122%	0.012	122%	116%
100	51	11.1 ± 0.50	102%	0.868	116%	104% (n.s.)
200	50	12.5 ± 0.49	110%	0.123	119%	106%
400	50	13.9 ± 0.89	122%	0.016	122%	108%
800	50	12.5 ± 0.28	107%	0.229	119%	108% (n.s)
**Chaga dose (μg/mL)**	
0	50	10.7 ± 0.50		
50	49	13.1 ± 0.60	122%	0.002	122%	113%
200	50	12.9 ± 0.55	121%	0.005	124%	117%
800	47	12.9 ± 0.65	121%	0.003	121%	116%

*Percent change in lifespan relative to control, significance from the representative survival curves shown in **Figures 1A–C**. The highest and lowest percent increase in lifespan relative to control in all replicates is also reported. Experiments were repeated in multiple independent trials (≥3), all with the same directional effect and similar magnitude of effect. Statistical significance (*p*-value) calculated with Kaplan–Meier log-rank test*.

Both lowbush cranberry (**Figure 1E**) and chaga (**Figure 1F**) treatments resulted in a bimodal response in lifespan extension. Two of the five selected lowbush cranberry treatment doses significantly increased lifespan: 50 and 400 μg/mL. Both 50 and 400 μg/mL lowbush cranberry treatments reliably, statistically increased lifespan up to 22% when compared to control. Treatment with 50, 200, and 800 μg/mL chaga extract significantly increased mean lifespan to 13.1 ± 0.6, 12.9 ± 0.5, and 12.9 ± 0.6 days, respectively, compared to a control of 10.7 ± 0.5 days in a representative trial (*p* < 0.05, Kaplan–Meier log-rank test). In addition to the effective chaga treatments doses shown in **Figure 1C**, we observed no significant change in mean or median lifespan at several additional doses (10, 20, and 100 μg/mL; **Figure 1F**). Crowberry treatment was also tested for lifespan extension at similar treatment doses as blueberry, but no significant effect was observed (Supplementary Figure S1).

Next, we investigated whether the lifespan-extending effects of blueberry, lowbush cranberry, and chaga were primarily due to secondary responses in *C. elegans* to an altered food source, OP50-1 *E. coli* exposed to the same treatment. OP50-1 *E. coli* growth in liquid culture was unaffected by the presence of Alaskan nutraceuticals at *C. elegans* lifespan-extending doses (data not shown). We also measured *C. elegans* lifespan on Alaskan berry and fungus treatments with UV irradiated OP50-1 *E. coli*. Both blueberry and lowbush cranberry treatments resulted in extended wild-type lifespan when compared to untreated control, even with UV-killed bacteria (*p* < 0.05; Kaplan–Meier log-rank test; Supplementary Figure S2). However, chaga treatment did not extend lifespan when *C. elegans* were fed UV-killed bacteria (0.09 < *p* < 0.4; Kaplan–Meier log-rank test).

### Alaskan Berry and Fungus Treatments Improve Healthspan

**FIGURE 2:**
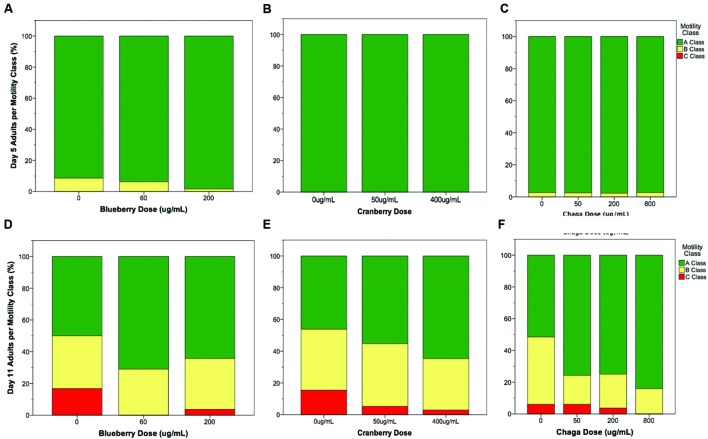
**Lifespan-extending Alaskan berry and fungus treatments improve wild-type *C. elegans* motility late in life**. Percent per motility class of middle age (day 5 adults; **A–C**) and old age (day 11 adults; **D–F**) for lifespan-extending Alaskan blueberry **(A,D)**, lowbush cranberry **(B,E)**, and chaga **(C,F)** are shown. Class A animals (green bars) moved normally and spontaneously, class B animals (yellow bars) moved abnormally and may have required prodding, and class C animals (red bars) were unable to translocate. *N* = 28–58 animals from at least three separate biological replicates per treatment group.

Interventions that increase lifespan do not necessarily increase health with age nor decrease the proportion of time spent living in a frail state (Bansal et al., 2015). To address this possibility with Alaskan berry and fungus treatments that extended lifespan (**Figure 1**), we studied several measures of healthspan. In *C. elegans*, the ability to move spontaneously and actively (i.e., motility) declines stochastically (Herndon et al., 2002). At mid-life (day 5 of adulthood), both control and lifespan-extending Alaskan berry and fungus treatments tested had nearly 100% normal motility, as expected (**Figures 2A–C**). However, all of the lifespan-extending Alaskan berry and fungus treatments maintained healthy motility well into late adulthood (day 11) by significantly increasing the ratio of normally, spontaneously moving adults (Class A) to abnormally, non-spontaneously moving (Class B) and frail, immobile (Class C) adults (*p* < 0.01 ordinal logistic model; **Figures 2D–F**). There were no dose-dependent effects on motility within blueberry and lowbush cranberry treatments (*p* > 0.2, ordinal logistic model). Then, 800 μg/mL chaga treatment significantly decreased the incidence of Class C adults at Day 11 of adulthood when compared to control, 50, and 200 μg/mL chaga (*p* < 0.001, ordinal logistic model).

**FIGURE 3:**
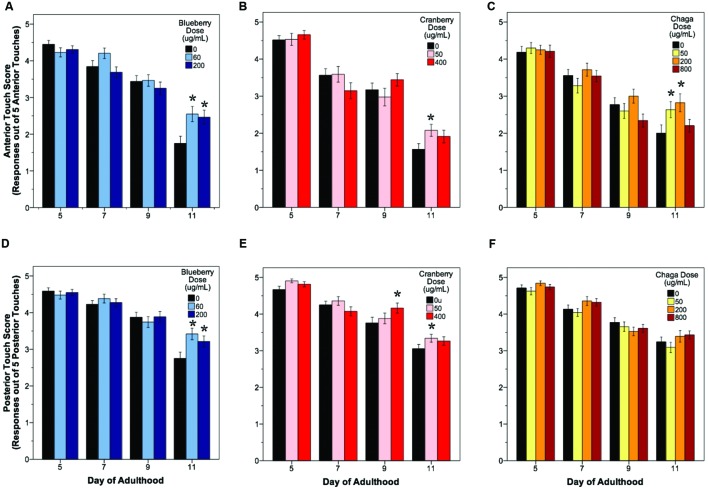
**Lifespan-extending Alaskan berry and fungus treatments improve wild-type *C. elegans* gentle touch response late in life**. Anterior **(A–C)** and posterior **(D–F)** mean touch score for each treatment group is shown. Asterisks denote significance from age-matched control (*p* < 0.05; Poisson log linear model). Bars represent mean ± standard error of the mean. *N* = 28–58 animals from at least three separate biological replicates per treatment group.

The ability to sense and respond to touch (i.e., touch response) also decreases with age in *C. elegans*. We observed a significant age-related decrease in both anterior and posterior touch response (i.e., the number of positive responses to five touches at the anterior or posterior end of the animal) in all control groups, as expected (**Figure 3**, black bars). Overall, lifespan-extending Alaskan berry and fungus treatments slowed this decline in mechanosensation by late adulthood (day 9 or day 11; **Figure 3**). Both blueberry treatments robustly improved anterior (1.75 ± 0.15 control vs. 2.5 ± 0.15 60 μg/mL and 2.4 ± 0.16 200 μg/mL; *p <* 0.02) and posterior touch response (2.7 ± 0.14 control vs. 3.4 ± 0.13 60 μg/mL and 3.2 ± 0.14 200 μg/mL; *p <* 0.02) at day 11 when compared to age-matched control. Low dose lowbush cranberry treatment (50 μg/mL) improved both anterior and posterior touch response over control at day 11 of adulthood (anterior: 1.7 ± 0.14 vs. 2.2 ± 0.18; posterior: 3.1 ± 0.11 vs. 3.3 ± 0.11; *p <* 0.05). The higher lowbush cranberry treatment (400 μg/mL) improved posterior touch response at day 9 of adulthood (3.75 ± 0.11 vs. 3.9 ± 0.11; *p <* 0.02), then dropped to control levels at day 11 (3.1 ± 0.11 vs. 3.26 ± 0.11; **Figure 3E**). Two of the three lifespan-extending chaga treatments improved touch response: both 50 (2.63 ± 0.18) and 200 μg/mL (2.82 ± 0.21) chaga treatments significantly improved anterior (but not posterior) touch response compared to control (2.0 ± 0.16) at day 11 (*p <* 0.05).

**FIGURE 4:**
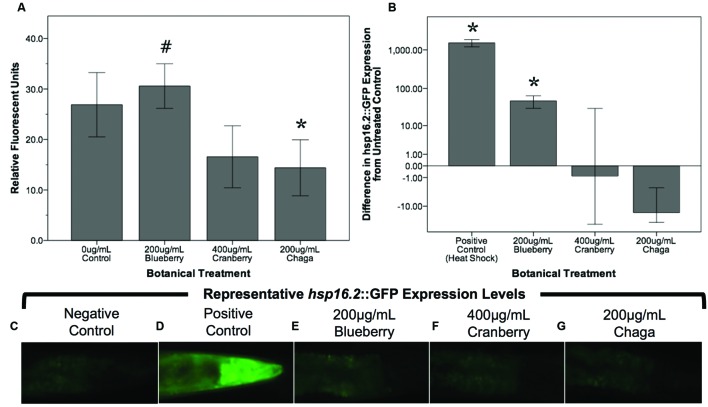
**Lifespan-extending Alaskan berry and fungus treatments change endogenous ROS levels in young wild-type *C. elegans***. **(A)** The change in endogenous ROS measured by DCF-DA after treating young adults for 48 h with selected lifespan-extending Alaskan berry and fungus treatments is shown. The percent change on a logarithmic scale of *hsp16.2*::GFP expression in the pharynx region of young adults treated for 48 h with selected berry and fungus treatments relative to untreated control **(B)** and representative images **(C–G)** are also shown. Note the extreme increase in *hsp16.2*::GFP expression in response to heat shock. Bars represent mean ± standard error of the mean of each replicate, each with two or three technical replicates for the DCF-DA assay. Hashtag (*p* = 0.085) and asterisks (*p* < 0.06; one-way ANOVA with Tukey *post hoc*) denote significance from 0 μg/mL control.

Reactive oxygen species are considered a biomarker of overall health and are associated with aging (Kregel and Zhang, 2007; Campos et al., 2014; Stuart et al., 2014). To test the hypothesis that lifespan-extending berry and fungus treatments decrease damaging ROS early in life, we measured ROS within whole, live young adult (day 2) *C. elegans* populations using H_2_DCF-DA. Blueberry, lowbush cranberry, and chaga treatments differentially influenced endogenous management of ROS (**Figure 4A**). Treatment with blueberry trended toward increasing ROS levels (113.7% of control; *p* = 0.085; One-way ANOVA) while chaga treatment strikingly decreased ROS (53.5% of control; *p* = 0.06; One-way ANOVA). Lowbush cranberry treatment had no effect on ROS early in adulthood when compared to control (*p* = 1.0; One-way ANOVA), although lowbush cranberry-mediated ROS levels were not significantly different from chaga treatments. Increased expression of the heat shock protein HSP-16.2 is a marker for ROS stress within *C. elegans*. To further describe ROS stress in young adults treated with lifespan-extending Alaskan berry and fungus treatments, we measured the *hsp-16.2*::GFP gene reporter fluorescence in the pharynx (**Figures 4B–G**). Blueberry treatment significantly increased *hsp-16.2*::GFP expression (146% of control; *p* < 0.05; One-way ANOVA). Both chaga and lowbush cranberry treatments did not significantly alter *hsp-16.2*::GFP gene expression (*p* > 0.2; One-way ANOVA).

Interventions that increase aging are often associated with a decrease in fecundity (Jafari and Rose, 2006). We found that lifespan-extending Alaskan berry and fungus treatments did not influence total progeny produced per individual (*p* > 0.7; One-way ANOVA; *N* = 24 per treatment; **Table 3**). When we considered the number of progeny produced per individual on specific days of adulthood, the blueberry treatments had numerically small (<15 progeny) but significant effects (*p* < 0.05; One-way ANOVA with Tukey *post hoc* analysis). However, no treatments extend the number of days individuals produced viable progeny (Supplementary Figure S3).

**Table 3:** Alaskan berry and fungus treatments do not alter total progeny produced by wild-type *C. elegans*.

Treatment		Total progeny produced per adult (mean ± SEM)
Blueberry dose (μg/ml)	0	190.7 ± 27.5
	60	198.2 ± 13.9
	200	195.2 ± 38.0
Lowbush cranberry dose (μg/ml)	0	183.4 ± 19.3
	50	201.7 ± 10.2
	400	198.3 ± 7.2
Chaga dose (μg/ml)	0	173.4 ± 7.47
	50	174.9 ± 14.7
	200	188.1 ± 4.6

*Experiments were repeated in three independent trials each with *N* = 8 parents per treatment group, all with no significant effect observed*.

### Alaskan Berry and Fungus Treatments Differentially Alter Mechanosensory Neuron Aging

**FIGURE 5:**
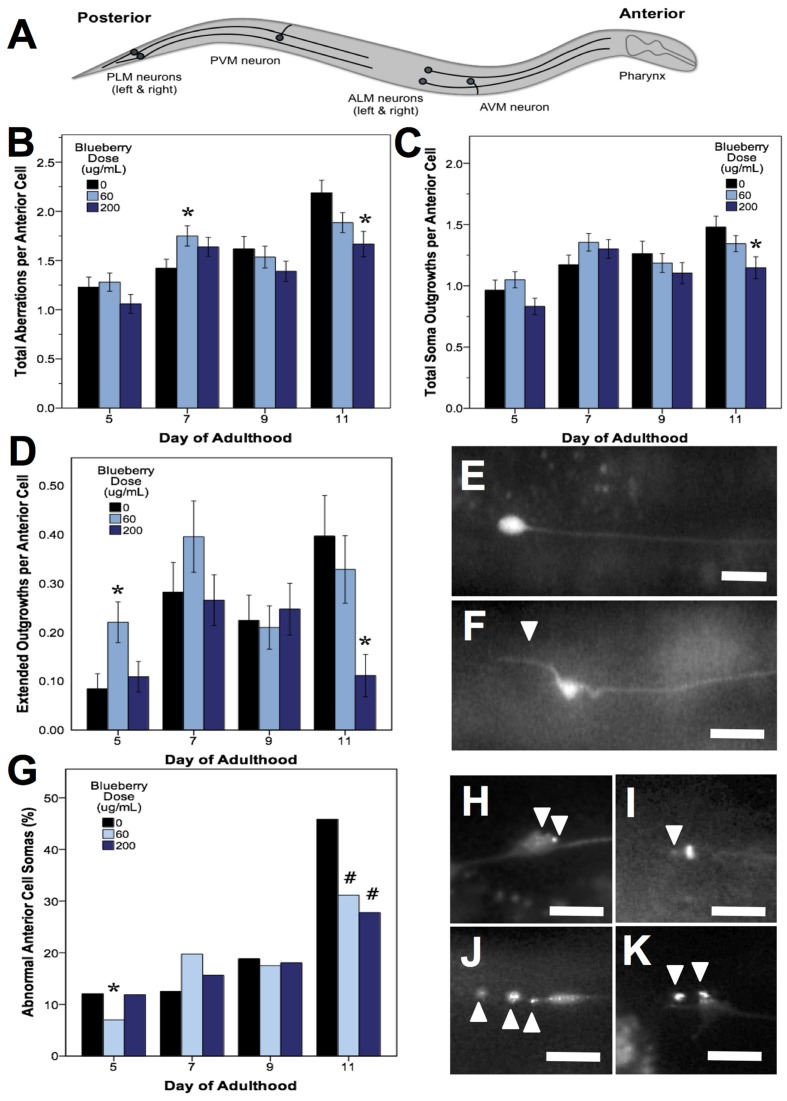
**Alaskan blueberry treatments alter the accumulation of aberrations in aging anterior touch receptor neurons**. Animals from control and lifespan-extending blueberry treatments (60 and 200 μg/mL) were imaged for touch receptor neuron aberrations at days 5, 7, 9, and 11 of adulthood. A schematic of the locations of the six GFP-labeled touch receptor neurons **(A)** is shown. Neuron aging markers in order of broad to more specific effects are shown: Mean number of total aberrations **(B)**, soma outgrowths (of all lengths) **(C)**, extended outgrowths (≥2× longer than soma diameter) per anterior (ALM) neuron **(D)** are shown along with representative images of ALM somas free of aberrations **(E)** and with extended outgrowth **(F)**. Percent of all anterior cells imaged with abnormal somas **(G)** is shown along with example ALM neurons with abnormal somas **(H–K)**. Bars represent mean ± standard error of the mean. Asterisks (*p* < 0.05) and hashtags (*p* < 0.08) denote significance from age-matched control. Nworms = 28–62 and Nanterior cells = 48–101 per bar. White arrowheads denote location of specific aberration. All images were collected at 20× magnification and were cropped to the same scale (no other image processing was performed). White scale bars are 10 μm.

*C. elegans* mechanosensory neurons sense soft touch and exhibit changes in morphology with age and with modifications in cellular signaling (Pan et al., 2011; Tank et al., 2011; Toth et al., 2012). To visualize these morphological changes with age, we used a *C. elegans* strain with GFP-labeled touch receptor neurons (*zdIs5* [mec-4p::GFP]). There are six *C. elegans* touch receptor neurons: two anterior lateral (ALML, ALMR), one anterior ventral (AVM), two posterior lateral (PLML, PLMR), and one posterior ventral (PVM), which are illustrated in **Figure 5A**. This study focused on the two pairs of lateral neurons because they are the most sensitive to change with age. The four lateral neurons (ALML, ALMR, PLML, and PLMR) are typified by a circular cell body (soma) and a single, straight process (representing the axon and dendrites) extending toward the anterior end of the animal. Posterior neurons have an additional outgrowth from the soma toward the posterior of the animal. We considered any morphological deviations from this norm to be “neuronal aberrations,” but did not assume that these aberrations were functionally deleterious.

**FIGURE 6:**
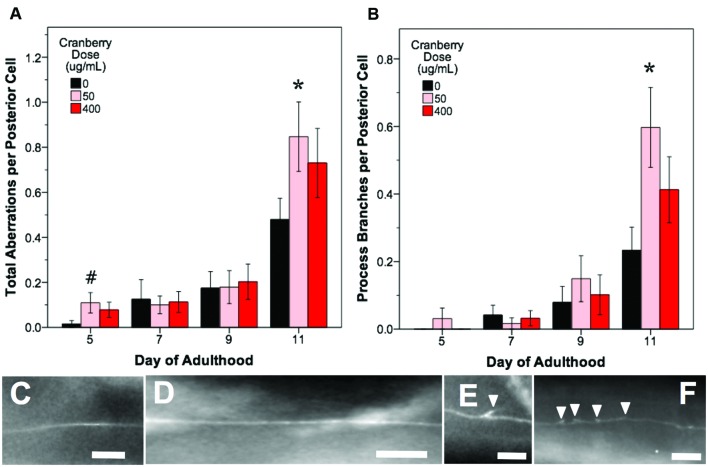
**Alaskan lowbush cranberry treatments increase occurrence of process branching in aging posterior touch receptor neurons**. Animals from control and lifespan-extending lowbush cranberry treatments (50 and 400 μg/mL) were imaged for touch receptor neuron aberrations at days 5, 7, 9, and 11 of adulthood. Mean number of total aberrations **(A)** and branches observed on the dentrite/axon per posterior lateral touch receptor (PLM) neuron **(B)** are shown. Representative images of posterior neuron processes with no aberrations **(C,D)**, with one process branch **(E)**, and with multiple process branching events **(F)** are also shown. Bars represent mean ± standard error of the mean. Asterisks (*p* < 0.05) and hashtags (*p* < 0.085) denote significance from age-matched control. Nposterior cells = 48–73 per bar. In all images, the anterior end of the animal is to the right and posterior lateral touch receptor (PLM) neurons are shown. All images were collected at 20× magnification and were cropped to the same scale (no other image processing was performed). White scale bars are 10 μm.

**FIGURE 7:**
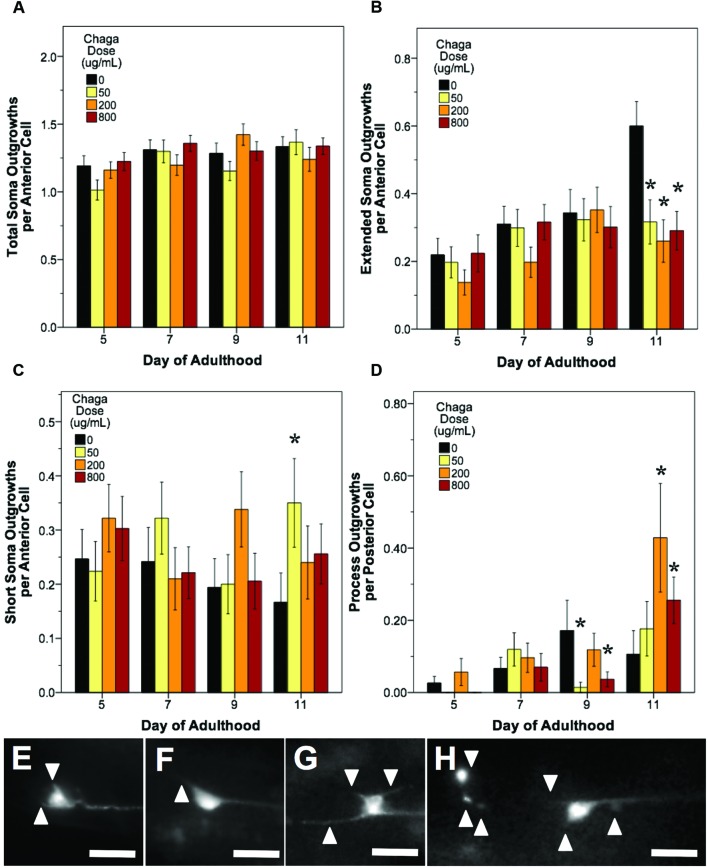
**Alaskan chaga treatments impact aging anterior and posterior touch receptor neurons**. Animals from control and lifespan-extending chaga treatments (50, 2, and 800 μg/mL) were imaged for touch receptor neuron aberrations at days 5, 7, 9, and 11 of adulthood. The total number of anterior cell soma outgrowths was unchanged with chaga treatment **(A)**, but the mean number of extended outgrowths (≥2× longer than soma diameter) **(B)** and short soma outgrowths (<1× soma diameter) per anterior cell **(C)** were impacted. Posterior cell process outgrowths **(D)** represents branching or loop phenotypes observed from the axon and/dendrites of the two posterior lateral neurons (PLM). Representative images of short soma outgrowths **(E)**, standard soma outgrowth (1× soma diameter; **F**), different length soma outgrowths **(G)** and extreme aberrations **(H)** are also shown. Bars represent mean ± standard error of the mean. Asterisks (*p* < 0.05) denote significance from age-matched control. Nworms = 28–50, Nanterior cells = 50–95, and Nposterior cells = 56–100 per bar. All images were collected at 20× magnification and were cropped to the same scale (no other image processing was performed). White scale bars are 10 μm.

In all treatments, we observed similar morphological changes with age in the untreated control groups, as expected (**Figures 5**–**7**; black bars). Notably, as they aged, all control populations exhibited significantly increased numbers of total aberrations per anterior cell (day 11 is 179% of day 5 levels; *p* < 0.05; Poisson log linear model; **Figure 5B**; black bars) and total aberrations per posterior cell (day 11 is 3154% of day 5 levels; *p* < 0.05; Poisson log linear model; **Figure 6A**; black bars). In anterior cells, the total number of extended outgrowths increased with age (day 11 is 469% of day 5 levels; *p* < 0.05; Poisson log linear model; **Figures 5D** and **7B**; black bars). In posterior cells, an increase in process branching was the primary driver for the increased total aberrations per cell (increased from no occurrences at day 5 to 23.2% occurrence at day 11; *p* < 0.05; Poisson log linear model; **Figure 6B**; black bars). These findings generally align with previously described age-related morphological changes observed in touch receptor neurons (Pan et al., 2011; Tank et al., 2011; Toth et al., 2012).

Blueberry treatments that significantly extended lifespan (60 and 200 μg/mL) decreased the total number of aberrations observed per animal relative to control by day 11 of adulthood (79.2 and 71.8% of control; *p* < 0.05; Poisson log linear model). At the cellular level, only anterior lateral neuron morphology was altered with blueberry treatment (86.2 and 76.2% of control; *p* < 0.05; Poisson log linear model; **Figure 5B**). The primary aberration driving this difference in anterior neuron morphology with blueberry treatment was soma outgrowths (90.9 and 77.6% of control levels; *p* < 0.05; Poisson log linear model; **Figure 5C**), specifically extended soma outgrowths (<2× soma diameter; **Figure 5D**). The percent of anterior cell somas with an abnormal shape or location significantly decreased with 200 μg/mL blueberry treatment day 11 of adulthood (67.4 and 56.5% of control; *p* < 0.05 Poisson log linear model; **Figure 5G**) and was the only other specific aberration significantly altered with blueberry treatment. Conversely, on day 5 of adulthood, low dose blueberry (60 μg/mL) treatment significantly increased extended outgrowths and significantly decreased percent cells with abnormal cell bodies (*p* < 0.05; Poisson log linear model), suggesting that a relationship between these two aberrations exists early in life. Posterior touch receptor neuron aging was unaffected by blueberry treatment (*p* > 0.8; Poisson log linear model).

In contrast to blueberry treatment, lowbush cranberry treatment increased only posterior cell aberrations (**Figure 6A**). Treatment with low dose lowbush cranberry (50 μg/mL) significantly increased total posterior cell aberrations both at mid-life (day 5; 719% of control) and old age (day 11; 177% of control; *p* < 0.01; Poisson log linear model). This increase in total aberrations was due solely to increased posterior cell process branching compared to control by day 11 of adulthood (**Figure 6B**). Anterior cell aberration development with age was unaffected by 50 μg/mL lowbush cranberry treatment (*p* > 0.8; Poisson log linear model). The higher lowbush cranberry treatment (400 μg/mL), which also increased lifespan, had no effect on age-related development of posterior cell aberrations (*p* > 0.1; Poisson log linear model). Then, 400 μg/mL lowbush cranberry treatment significantly decreased the number of anterior cell aberrations by day 11 of adulthood (83.1% of control; *p* < 0.05; Poisson log linear model), but we are unable to relate this decrease to any specific type of neuronal aberration.

Treatment with lifespan-extending chaga doses elicited effects similar to both blueberry and lowbush cranberry treatments: both anterior cell soma outgrowths and posterior cell process outgrowth events were impacted (**Figure 7**). While chaga treatment did not significantly alter the accumulation of total anterior cell outgrowths with age (*p* > 0.8; Poisson log linear model; **Figure 7A**), these treatments did affect the lengths of the soma outgrowths observed. In all three chaga treatments, the occurrence of extended outgrowths (≥2× soma diameter) significantly decreased compared to control at day 11 of adulthood (43.3–52.8% of control; *p* < 0.05; Poisson log linear model; **Figure 7B**). Conversely, short soma outgrowths (<1× soma diameter) increased by day 11 with chaga treatment (153–210% of control; *p* < 0.05; Poisson log linear model; **Figure 7C**). Posterior cell process outgrowths, which consist of both straight branches pointing away from the process and branches that loop back to the process (i.e., loops), were affected by all three chaga treatments later in life (166–403% of control; *p* < 0.05; Poisson log linear model; **Figure 7D**). Specifically, treatment with low dose chaga (50 μg/mL) significantly reduced posterior cell process outgrowths at day 9 of adulthood (8.3% of control; *p* < 0.05; Poisson log linear model), but these aberrations rose to control levels at day 11 (*p* > 0.2; Poisson log linear model). Treatment with 200 μg/mL chaga quadruples the occurrence of posterior cell process outgrowths at day 11 of adulthood compared to control (403% of control; *p* < 0.05; Poisson log linear model). High dose chaga treatment (800 μg/mL) caused a significant decrease in posterior cell process outgrowths at day 9 of adulthood compared to control and a significant increase by day 11 (241% of control; *p* < 0.05; Poisson log linear model).

## Discussion

**FIGURE 8:**
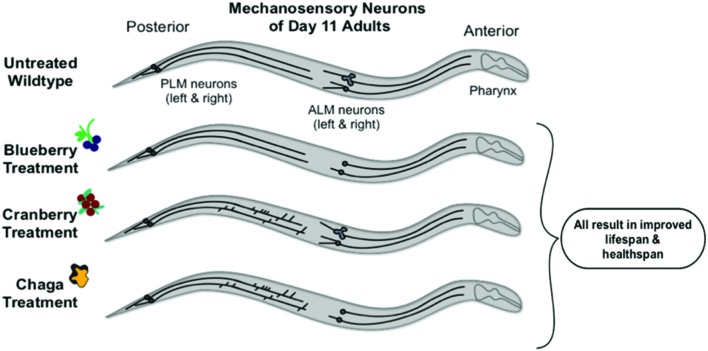
**Schematic summary of results**. Non-treated wild-type aging *C. elegans* touch receptor neurons exhibit increased extended outgrowths from the anterior neuron (ALM) soma and increased abnormal ALM soma by late life (day 11 of adulthood). Alaskan berry and fungus treatments impact this neuron aging trajectory by blocking accumulation of ALM aberrations (i.e., blueberry), increasing the incidence of posterior neuron (PLM) aberrations (i.e., lowbush cranberry), or both (i.e., chaga) when compared to age-matched controls. Nevertheless, all three treatments significantly increase lifespan and improve markers of healthspan (i.e., motility and touch response late in life).

Compared to the modern “Western” lifestyle, traditional North American Native and subsistence lifestyles are associated with lower incidence of age-related and chronic disorders, particularly cardiovascular disease (Ebbesson et al., 2005; Mohatt et al., 2007). Differences in the diet of these two lifestyles are proposed factors for the improved health. To further explore the influence of traditional food sources on the functional aging process, we tested the effects of Alaskan berry and fungus extracts on *C. elegans* lifespan, healthspan, and markers of touch receptor neuron aging. Alaskan blueberry, lowbush cranberry, and chaga treatment increased lifespan at varying doses (i.e., 50–800 μg/mL; **Figure 1** and **Table 2**). Increased lifespan may be uncoupled from improved health in old age (e.g., healthspan), thus measuring length of life alone does not necessarily provide an accurate portrayal of aging (Bansal et al., 2015). We find that measures of healthspan are either improved (i.e., motility, touch response; **Figures 2** and **3**) or not affected (i.e., total viable progeny produced; **Table 3**) throughout the lifespan of animals treated with Alaskan berries and fungus. Each of the Alaskan berry and fungus treatments resulted in varied touch receptor neuron aging trajectories, with both decreased incidence of anterior neuron (ALM) soma outgrowths and increased incidence of posterior neuron (PLM) process branching (**Figure 8**). Nonetheless, both changes correlated with improved touch response (**Figures 3** and **5**–**8**). Importantly, this suggests that the development of specific touch receptor neuron morphologies impact neuronal and organismal function differently and reflects the importance of examining multiple healthspan markers in aging studies. These findings demonstrate that beneficial nutritional lifespan interventions differentially impact touch receptor neuron aging and, thus, support the body of research describing numerous cellular strategies leading to increased lifespan and improved health (reviewed in Lopez-Otin et al., 2013).

Alaskan blueberry treatment effects are distinct from the other treatments; blueberry elicited the greatest increase in lifespan (up to 47%) and improved both anterior and posterior touch response late in life, while only impacting ALM morphology (i.e., decreased extended soma outgrowths and incidence of abnormal cell bodies; **Figure 5**). There is also a time-dependent and dose-dependent response to blueberry treatment that is unique from the other Alaskan nutraceutical treatments. First, middle-aged adults (day 5) treated with 60 μg/mL blueberry exhibit a touch receptor neuron phenotype associated with decreased health later in life (i.e., increased incidence of extended outgrowths from anterior cell somas; **Figure 5D**) and no improvement in other markers of healthspan (i.e., motility, **Figure 2A**; touch response, **Figures 3A,D**). Second, the higher 200 μg/mL treatment elicited a significant decrease in extended outgrowths by late life (day 11), while 60 μg/mL did not (**Figure 5D**). Also, in young adults (day 2) treated with 200 μg/mL blueberry, we observed increased levels of damaging ROS within live animals and increased *hsp-16.2*::GFP expression, a marker of ROS stress when compared to controls (**Figure 4**). Importantly, heat-shock treatments, which induce *hsp-16.2*::GFP expression, are known to result in improved stress response via hormetic mechanisms (Link et al., 1999). Blueberry treatment (200 μg/mL) also slightly, but significantly, decreased the number of progeny produced on day 2 of adulthood (27 ± 13.1 fewer progeny than control; Supplementary Figure S3) but did not impact total progeny produced over reproductive lifespan; again suggesting that early in life, blueberry treatment induces stress-response signaling.

These dose- and time-dependent results indicate an involvement of two interesting biological phenomenon in the blueberry treatment effects: hormesis and xenohormesis. Hormesis describes the dose-dependent effect wherein low doses of a treatment elicit a beneficial response while high doses of the same treatment are elicit a negative response or are toxic. Xenohormesis is the adaptive, defensive response of animals to consuming secondary metabolites produced by environmentally stressed plants (e.g., polyphenols; Howitz and Sinclair, 2008). Xenohormesis may also be described as one organism (i.e., the consumer) benefiting from the stress response, or hormesis, of another organism (i.e., the plant). This intra-species signaling is proposed to activate stress-response signaling in animals via interactions with signaling molecules (as opposed to general antioxidant effects), which improves the health and survival of the consumer (Howitz and Sinclair, 2008). Xenohormesis may be involved in the blueberry treatment effects because of the increased *C. elegans* lifespan following the consumption of Alaskan blueberry extract, which is known to have elevated polyphenolic content relative to other blueberry species (Grace et al., 2014), indicative of UV-stress. Additionally, the observation of a detrimental neuronal phenotype coupled with increased markers of ROS stress and decreased progeny early in life followed by improved lifespan, healthspan, and touch receptor neuron health late in life suggests that hormesis within the worm is likely also occurring.

Others have proposed that polyphenols, including extracts from blueberry, exert their effects on stress-response and longevity through mechanisms other than antioxidant activity, such as hormesis and xenohormesis. For example, Elks et al. (2011) observed that feeding a hypertensive rat model a blueberry-enriched diet improved renoprotection and oxidative stress markers in the long term (6 or 12 weeks) but *increased* oxidative stress in the short term (2 days). These authors also proposed xenohormesis as a mechanism of blueberry treatment effects. Additionally, Wilson et al. (2006) found that treatment with non-Alaskan, wild lowbush blueberry polyphenols not only increased lifespan but also improved response to heat stress and thermotolerance, processes that are markers of an adaptive response to a stressor (Gems and Partridge, 2008). Blueberry extracts have also been shown to modulate biological responses (e.g., inhibition of inflammation) at treatment doses low enough to preclude the action of antioxidants as ROS scavengers as the sole explanation of their effects in neuronal cell culture (Gustafson et al., 2012). Interactions of specific polyphenolic compounds with diverse cellular signaling pathways outside of antioxidant signaling involved in aging have also been described (e.g., quercitin from apples and insulin signaling; Youl et al., 2010). These results and previous examples suggest that blueberry treatment elicits health benefits through signaling mechanisms outside, or at least in addition to, antioxidant signaling and scavenging.

Lowbush cranberry treatment increased lifespan to a lower extent than blueberry (22% compared to 30–47%), improved both anterior and posterior touch response late in life, and only impacted PLM neuron aging (i.e., increased posterior process branching). Chaga treatment resulted in bimodal lifespan extension to a similar magnitude as lowbush cranberry (up to 24%), only improved anterior touch response, and both decreased extended ALM soma outgrowths and increased PLM process branching and loop events late in life. Interestingly, there is not a clear relationship between total phenolic, flavonoid, and anthocyanin contents and these lifespan, healthspan, and neuron aging results (**Table 1**), suggesting that the concentrations of these compounds alone is not enough to predict anti-aging bioactivity. Although both lowbush cranberry and chaga treatments resulted in a similar magnitude of lifespan extension, the lowbush cranberry extract contained nearly 3×, 7×, and 29× more total phenolic, flavonoid, and anthocyanin content, respectively, than the chaga extract. Also, the extract containing the highest levels of all three polyphenols, crowberry, did not extend the lifespan of wild-type animals at the doses tested. This lack of lifespan extension may be due to either testing doses with too high of polyphenolic content or the presence of competing, non-beneficial molecules in the crowberry extract that are not in the blueberry or lowbush cranberry extracts.

Chaga extract contained the lowest polyphenol levels of the studied extracts, yet, endogenous ROS levels of *C. elegans* following consumption of chaga extract support previous studies demonstrating that chaga extracts exert potent antioxidant effects (**Figure 4**) (Cui et al., 2005; Giridharan et al., 2011). While chaga contains polyphenolic compounds (Lee et al., 2007), the fungus also contains unique non-polyphenolic bioactive secondary compounds, such as steroid derivatives. Ergosterol peroxide, a steroid derivative unique to fungi, lichens, yeast, and sponges, was recently shown to have anti-cancer activity in a model of colorectal cancer (Kang et al., 2015). The low polyphenolic content measured in this study’s chaga extract, when compared to berry extracts coupled with the observed beneficial effects on aging (e.g., decreased ROS and increased lifespan), suggests that such a compound may be central to the health benefits of chaga. The lack of correlation between the polyphenolic content in these Alaskan nutraceutical extracts and ROS levels with the observed effects on whole animal and neuron aging suggests that the general antioxidant effect (i.e., removal of ROS) by these compounds may not be central to their involvement in healthy neuron aging. Rather, chaga-induced healthy neuron aging may be due to the interaction of polyphenolic and non-polyphenolic compounds with cellular signaling pathways distinct from antioxidant signaling pathways, another potential example of xenohormesis.

Anterior and posterior mechanosensory neurons respond to aging with different morphological changes: ALM neurons exhibit increased soma outgrowths and abnormal soma shapes while PLM neurons exhibit increased process outgrowths with age (i.e., branching, loops; Pan et al., 2011; Tank et al., 2011; Toth et al., 2012; Vayndorf et al., 2016). The heterogeneity in morphological responses to aging between ALM and PLM neurons may be due to their particular neural networks and, thus, activity levels. In general terms, the touch receptor neurons (including the pairs of ALM and PLM neurons) synapse on interneurons, which then synapse on motor neurons, resulting in forward or backward movement (reviewed in Goodman, 2006). As they age, the three anterior touch receptor neurons (ALML, ALMR, and AVM) form their own neural network via gap junctions, a characteristic not shared with the posterior touch receptor neurons (Chalfie et al., 1985). Additionally, only ALM neurons (not PLM) sensitize to high salt, hypoxia, dauer, and prolonged vibration, which prevents the animal from repeatedly changing direction in response to non-localized stressors (Chen and Chalfie, 2014). Toth et al. (2012) proposed that the age-related morphological differences between neurons of the same class (e.g., PLM and ALM neurons) and between classes of neurons (e.g., touch receptor versus dopamine neurons) may also be due to neuronal position (i.e., space to grow) or cell-specific gene expression. Taken together, the functional differences in the ALM and PLM neurons likely have consequences for their age-related morphological changes and, thus, their responses to the aging process.

We found that both *decreased* outgrowth from anterior neuron somas (i.e., soma outgrowths) and *increased* outgrowth from posterior neuron axons (i.e., process branching) late in life are associated with improved mechanosensory neuron function (i.e., touch response), improved motility, and increased lifespan (**Figure 8**). Thus, increased incidence of these age-related morphologies is not directly correlated with poorer health: both decreased incidence of ALM aberrations (i.e., soma outgrowths, abnormal soma shape) and increased incidence of PLM aberrations (i.e., process branching and loops) correlate with increased lifespan, improved motility, and improved touch response. Others have shown that specific genetic mutations known to be involved in aging (e.g., insulin signaling) seem to regulate specific mechanosensory neuron morphologies (Pan et al., 2011; Tank et al., 2011; Toth et al., 2012; Scerbak et al., 2014). Because of the apparent different cellular controls of these morphological changes, a synergy in maintaining organismal health with age may exist between the anterior and posterior neurons. Aspects of this potential synergy are evident in the treatment effects of chaga, which impacted both ALM and PLM neurons: although chaga treatment was not the most effective at extending lifespan at the doses tested (**Figure 1**), it maintained motility the best out of treatments tested (**Figure 2**) and maintained anterior touch response better than cranberry (**Figure 3**). What remains to be determined is whether increased incidence of morphological aberrations (e.g., PLM process branching) is a stimulatory response that *actively promotes* healthy aging or whether this phenotype occurs as a *consequence* of good health and aging. The ALM and PLM pairs of touch receptor neurons consistently exhibit differences in their aging trajectories; reflecting the multifaceted controls of aging at the cellular level.

## Conclusion

These data and conclusions also bring up an exciting aspect of studying the impacts of medicinal foods on aging: the opportunity to study biological complexities that can rarely be fully recapitulated by single genetic manipulations. The varied anti-aging impacts of these medicinal foods on the nervous system of *C. elegans* suggest that they may ameliorate chronic disorder development and progression, such as neurodegenerative diseases (e.g., Alzheimer’s and Parkinson’s diseases). Studying the impact of culturally valued foods, such as Alaskan berries and fungi, not only supports the traditional ecological knowledge that these foods are beneficial to health, but provides novel insights into the mechanisms of neuronal aging and aging in general.

## Author Contributions

CS conducted experiments, performed data analyses, and lead manuscript writing efforts. EV advised and assisted in experimental design and participated in in-depth discussions about methods, data analysis, and results. AH assisted in data collection and analysis. CM advised and assisted with Alaskan berry and fungus extractions. BT participated in in-depth methods and results discussions, provided detailed writing and editing assistance, and provided funding for the project.

## Conflict of Interest Statement

The authors declare that the research was conducted in the absence of any commercial or financial relationships that could be construed as a potential conflict of interest.

## References

[B1] Bansal A. Zhu L. J. Yen K. Tissenbaum H. A. (2015). Uncoupling lifespan and healthspan in *Caenorhabditis elegans* longevity mutants. *Proc. Natl. Acad. Sci. U.S.A.* 122 E277–E286. 10.1073/pnas.1412192112 25561524 PMC4311797

[B2] Bersamin A. Zidenberg-Cherr S. Stern J. Luick B. (2007). Nutrient intakes are associated with adherence to a traditional diet among Yup‘ik Eskimos living in remote Alaska Native communities: the CANHR Study. *Int. J. Circumpolar Health* 66 62–70. 10.3402/ijch.v66i1.18228 17451135

[B3] Bishop N. A. Lu T. Yankner B. A. (2010). Neural mechanisms of ageing and cognitive decline. *Nature* 464 529–535. 10.1038/nature08983 20336135 PMC2927852

[B4] Braeckman B. P. Houthoofd K. De Vreese A. Vanfleteren J. R. (2002). Assaying metabolic activity in ageing *Caenorhabditis elegans*. *Mech. Ageing Dev.* 123 105–119. 10.1016/S0047-6374(01)00331-1 11718805

[B5] Brenner S. (1974). The genetics of *Caenorhabditis elegans*. *Genetics* 77 71–94. 4366476 10.1093/genetics/77.1.71 PMC1213120

[B6] Campos P. Paulsen B. Rehen S. (2014). Accelerating neuronal aging in in vitro model brain disorders: a focus on reactive oxygen species. *Front. Aging Neurosci.* 6:292 10.3389/fnagi.2014.00292 PMC4209886 25386139

[B7] Chalfie M. Sulston J. E. White J. G. Southgate E. Thomson N. Brenner S. (1985). The neural circuit for touch sensitivity in *Caenorhabditis elegans*. *J. Neurosci.* 5 956–964. 3981252 10.1523/JNEUROSCI.05-04-00956.1985 PMC6565008

[B8] Chen X. Chalfie M. (2014). Modulation of *C. elegans* touch sensitivity is integrated at multiple levels. *J. Neurosci.* 34 6522–6536. 10.1523/JNEUROSCI.0022-14.2014 24806678 PMC4012311

[B9] Cui Y. Kim D. S. Park K. C. (2005). Antioxidant effect of *Inonotus obliquus*. *J. Ethnopharmacol.* 96 79–85. 10.1016/j.jep.2004.08.037 15588653

[B10] Dinstel R. Cascio J. Koukel S. (2013). The antioxidant level of Alaska’s wild berries: high, higher and highest. *Int. J. Circumpolar Health* 72:e2188 10.3402/ijch.v72i0.21188 PMC3751288 23977647

[B11] Ebbesson S. Adler A. Risica P. Ebbesson L. Yeh J.-L. Go O. (2005). Cardiovascular disease and risk factors in three Alaskan Eskimo populations: the Alaska-Siberia project. *Int. J. Circumpolar Health* 64 365–386. 10.3402/ijch.v64i4.18014 16277121

[B12] Elks C. M. Francis J. Stull A. Cefalu W. Shukitt-Hale B. Ingram D. (2013). “Overview of the Health Properties of Blueberries,” in *Bioactives in Fruit: Health Benefits and Functional Foods*, eds Skinner M. Hunter D. (Oxford: John Wiley & Sons), 251–271.

[B13] Elks C. M. Reed S. D. Mariappan N. Shukitt-Hale B. Joseph J. A. Ingram D. K. (2011). A blueberry-enriched diet attenuates nephropathy in a rat model of hypertension via reduction in oxidative stress. *PLoS ONE* 6:e24028 10.1371/journal.pone.0024028 PMC3174132 21949690

[B14] Gems D. Partridge L. (2008). Stress-response hormesis and aging: “That which does not kill us makes us stronger.” *Cell Metab.* 7 200–203. 10.1016/j.cmet.2008.01.001 18316025

[B15] Giridharan V. Thandavarayan R. Konishi T. (2011). Amelioration of scopolamine induced cognitive dysfunction and oxidative stress by *Inonotus obliquus* – a medicinal mushroom. *Food Funct.* 2 320–327. 10.1039/c1fo10037h 21779570

[B16] Goodman M. B. (2006). “Mechanosensation,” in *WormBook*, ed. The *C. elegans* Research Community and WormBook. Available at: http://www.wormbook.org (accessed January 06 2006). 10.1895/wormbook.1.62.1 PMC2806189 18050466

[B17] Grace M. H. Esposito D. Dunlap K. L. Lila M. A. (2014). Comparative analysis of phenolic content and profile, antioxidant capacity, and anti-inflammatory bioactivity in wild Alaskan and commercial *Vaccinium* berries. *J. Agric. Food Chem.* 62 4007–4017. 10.1021/jf403810y 24219831 PMC4026347

[B18] Guha S. Cao M. Kane R. Savino A. Zou S. Dong Y. (2012). The longevity effect of cranberry extract in *Caenorhabditis elegans* is modulated by daf-16 and osr-1. *Age (Dordr.)* 35 1559–1574. 10.1007/s11357-012-9459-x 22864793 PMC3776105

[B19] Gustafson S. J. Dunlap K. L. McGill C. M. Kuhn T. B. (2012). A nonpolar blueberry fraction blunts NADPH oxidase activation in neuronal cells exposed to tumer necrosis factor-α. *Oxid. Med. Cell Longev.* 12 1–12. 10.1155/2012/768101 PMC3317020 22530077

[B20] Halliwell B. Whiteman M. (2004). Measuring reactive species and oxidative damage in vivo and in cell culture: how should you do it and what do the results mean? *Br. J. Pharmacol.* 142 231–255. 10.1038/sj.bjp.0705776 15155533 PMC1574951

[B21] Herald T. Gadgil P. Tilley M. (2012). High-throughput microplate assays for screening flavonoid content and DPPH-scavenging activity in sorghum bran and flour. *J. Sci. Food Agric.* 92 2326–2331. 10.1002/jsfa.5633 22419130

[B22] Herndon L. Schmeissner P. Dudaronek J. Brown P. Listner K. Sakano Y. (2002). Stochastic and genetic factors influence tissue-specific decline in ageing *C. elegans*. *Nature* 419 808–814. 10.1038/nature01135 12397350

[B23] Howell A. Kalt W. Duy J. C. Forney C. F. (2001). Horticultural factors affecting antioxidant capacity of blueberries and other small fruit. *Horitechnology* 11 523–528.

[B24] Howitz K. T. Sinclair D. A. (2008). Xenohormesis: sensing the chemical cues of other species. *Cell* 133 387–391. 10.1016/j.cell.2008.04.019 18455976 PMC2504011

[B25] Iriti M. Vitalini S. Fico G. Faoro F. (2010). Neuroprotective herbs and foods from different traditional medicines and diets. *Molecules* 15 3517–3555. 10.3390/molecules15053517 20657497 PMC6263339

[B26] Jafari M. Rose M. (2006). Rules for the use of model organisms in antiaging pharmacology. *Aging Cell* 5 17–22. 10.1111/j.1474-9726.2006.00195.x 16441839

[B27] Joseph J. Cole G. Head E. Ingram D. (2009). Nutrition, brain aging, and neurodegeneration. *J. Neurosci.* 29 12795–12801. 10.1523/JNEUROSCI.3520-09.2009 19828791 PMC6665319

[B28] Joseph J. A. Shukitt-Hale B. Denisova N. A. (1999). Reversals of age-related declines in neuronal signal transduction, cognitive, and motor behavioral deficits with blueberry, spinach, or strawberry dietary supplementation. *J. Neurosci.* 19 8114–8121. 10479711 10.1523/JNEUROSCI.19-18-08114.1999 PMC6782471

[B29] Joseph S. Edirisinghe I. Burton-Freeman B. (2014). Berries: anti-inflammatory effects in humans. *J. Agric. Food Chem.* 62 3886–3903. 10.1021/jf4044056 24512603

[B30] Kalt W. Ryan D. A. Duy J. C. Prior R. L. Ehlenfeldt M. K. Kloet S. P. V. (2001). Interspecific variation in anthocyanins, phenolics, and antioxidant capacity among genotypes of highbush and lowbush blueberries (*Vaccinium* section cyanococcus spp.). *J. Agric. Food Chem.* 49 4761–4767. 10.1021/jf010653e 11600018

[B31] Kang J. H. Jang J. E. Mishra S. K. Lee H. J. Nho C. W. Shin D. (2015). Ergosterol peroxide from chaga mushroom (*Inonotus obliquus*) exhibits anti-cancer activity by down-regulation of the B-catenin pathway in coloresctal cancer. *J. Ethnopharmacol.* 173 303–312. 10.1016/j.jep.2015.07.030 26210065

[B32] Kim H. Song M. J. (2014). Analysis of traditional knowledge for wild edible mushrooms consumed by residents living in Jirisan National Park (Korea). *J. Ethnopharmacol.* 153 90–97. 10.1016/j.jep.2013.12.041 24445190

[B33] Kregel K. Zhang H. (2007). An integrated view of oxidative stress in aging: basic mechanisms, functional effects, and pathological considerations. *Am. J. Physiol.* 292 R18–R36. 10.1152/ajpregu.00327.2006 16917020

[B34] Krikorian R. Shidler M. Nash T. Kalt W. Vinqvist-Tymchuk M. Shukitt-Hale B. (2010). Blueberry supplementation improves memory in older adults. *J. Agric. Food Chem.* 58 3996–4000. 10.1021/jf9029332 20047325 PMC2850944

[B35] Lee I. K. Kim Y. S. Jang Y. W. Jung J. Y. Yun B. S. (2007). New antioxidant polyphenols from the medicinal mushroom *Inonotus obliquus*. *Bioorg. Med. Chem. Lett.* 17 6678–6681. 10.1016/j.bmcl.2007.10.072 17980585

[B36] Link C. D. Cypser J. R. Johnson C. J. Johnson T. E. (1999). Direct observation of stress resonse in *Caenorhabditis elegans* using a reporter transgene. *Biol. Sci. Med. Sci.* 57 B109–B114. 10.1379/1466-1268(1999)004<0235:doosri>2.3.co;2 PMC312938 10590837

[B37] Liu Z. Schwimer J. Liu D. Greenway F. Anthony C. Woltering E. (2005). Black raspberry extract and fractions contain angiogenesis inhibitors. *J. Agric. Food Chem.* 53 3909–3915. 10.1021/jf048585u 15884816

[B38] Lohachoompol V. Srzednicki G. Craske J. (2004). The change of total anthocyanins in blueberries and their antioxidant effect after drying and freezing. *Biomed. Res. Int.* 2004 248–252. 10.1155/S1110724304406123 PMC1082901 15577185

[B39] Lopez-Otin C. Blasco M. A. Partridge L. Serrano M. Kroemer G. (2013). The hallmarks of aging. *Cell* 153 1194–1217. 10.1016/j.cell.2013.05.039 23746838 PMC3836174

[B40] Loring P. Gerlach S. C. (2009). Food, culture, and human health in Alaska: an integrative health approach to food security. *Environ. Sci. Policy* 12 466–478. 10.1016/j.envsci.2008.10.006

[B41] Miller M. Shukitt-Hale B. (2012). Berry fruit enhances beneficial signaling in the brain. *J. Agric. Food Chem.* 60 5709–5715. 10.1021/jf2036033 22264107

[B42] Mohatt G. Plaetke R. Klejka J. Luick B. Lardon C. Bersamin A. (2007). The Center for Alaska Native Health Research Study: a community-based participatory research study of obesity and chronic disease-related protective and risk factors. *Int. J. Circumpolar Health* 66 8–18. 10.3402/ijch.v66i1.18219 17451130

[B43] Ogawa K. Sakakibara H. Iwata R. Ishii T. Sato T. Goda T. (2008). Anthocyanin composition and antioxidant activity of the crowberry (*Empetrum nigrum*) and other berries. *J. Agric. Food Chem.* 56 4457–4462. 10.1021/jf800406v 18522397

[B44] Pan C. L. Peng C. Y. Chen C. H. McIntire S. (2011). Genetic analysis of age-dependent defects of the *Caenorhabditis elegans* touch receptor neurons. *Proc. Natl. Acad. Sci. U.S.A.* 108 9274–9279. 10.1073/pnas.1011711108 21571636 PMC3107274

[B45] Rea S. L. Wu D. Cypser J. R. Vaupel J. W. Johnson T. E. (2005). A stress-sensitive reporter predicts longevity in isogenic populations of *Caenorhabditis elegans*. *Nat Genet.* 37 894–898. 10.1038/ng1608 16041374 PMC1479894

[B46] Scerbak C. (2016). *Modulating Neuronal Aging: Insights from Insulin Signaling Genes and Alaskan nutraceuticals*. Ph.D. thesis, University of Alaska Fairbanks, Fairbanks, AK.

[B47] Scerbak C. Vayndorf E. Parker A. Neri C. Driscoll M. Taylor B. (2014). Insulin signaling in the aging of healthy and proteotoxically stressed mechanosensory neurons. *Front. Genet.* 5:212 10.3389/fgene.2014.00212 PMC4107846 25101108

[B48] Schulz T. Zarse K. Voigt A. Urban N. Birringer M. Ristow M. (2006). Glucose restriction extends *Caenorhabditis elegans* life span by inducing mitochondrial respiration and increasing oxidative stress. *Cell Metab.* 6 280–293. 10.1016/j.cmet.2007.08.011 17908557

[B49] Shukitt-Hale B. (2012). Blueberries and neuronal aging. *Gerontology* 58 518–523. 10.1159/000341101 22907211

[B50] Song B. Sapper T. Burtch C. Brimmer K. Goldschmidt M. Ferruzzi M. (2013). Photo- and thermodegradation of anthocyanins from grape and purple sweet potato in model beverage systems. *J. Agric. Food Chem.* 61 1364–1372. 10.1021/jf3044007 23330879

[B51] Spencer J. (2008). Food for thought: the role of dietary flavonoids in enhancing human memory, learning and neuro-cognitive performance. *Proc. Nutr. Soc.* 67 238–252. 10.1017/S0029665108007088 18412998

[B52] Stuart J. Maddalena L. Merilovich M. Robb E. (2014). A midlife crisis for the mitochondrial free radical theory of aging. *Longev. Healthspan* 3 4 10.1186/2046-2395-3-4 PMC3977679 24690218

[B53] Tank E. Rodgers K. Kenyon C. (2011). Spontaneous age-related neurite branching in *Caenorhabditis elegans*. *J. Neurosci.* 31 9279–9288. 10.1523/JNEUROSCI.6606-10.2011 21697377 PMC3148144

[B54] Toth M. Melentijevic I. Shah L. Bhatia A. Lu K. Talwar A. (2012). Neurite sprouting and synapse deterioration in the aging *Caenorhabditis elegans* nervous system. *J. Neurosci.* 32 8778–8790. 10.1523/JNEUROSCI.1494-11.2012 22745480 PMC3427745

[B55] Vayndorf E. M. Scerbak C. Hunter S. Neuswanger J. R. Toth M. Parker A. J. (2016). Morphological remodeling of *C. elegans* neurons during aging is modified by compromised protein homeostasis. *NPJ Aging Mech. Dis.* 2 16001 10.1038/npjamd.2016.1 PMC4920063 27347427

[B56] Whyte A. Schafer G. Williams C. (2015). Cognitive effects following acute wild blueberry supplementation in 7- to 10-year-old children. *Eur. J. Nutr.* 10.1007/s00394-015-1029-4 [Epub ahead of print]. 26437830

[B57] Wiegant F. Surinova S. Ytsma E. Langelaar-Makkinje M. Wikman G. Post J. A. (2008). Plant adaptogens increase lifespan and stress resistance in *C. elegans*. *Biogerontology* 10 27–42. 10.1007/s10522-008-9151-9 18536978

[B58] Wilson M. A. Shukitt-Hale B. Kalt W. Ingram D. K. Joseph J. A. Wolkow C. A. (2006). Blueberry polyphenols increase lifespan and thermotolerance in *Caenorhabditis elegans*. *Aging Cell* 5 59–68. 10.1111/j.1474-9726.2006.00192.x 16441844 PMC1413581

[B59] Wink M. (2003). Evolution of secondary metabolites from an ecological and molecular phylogenetic perspective. *Phytochemistry* 64 3–19. 10.1016/S0031-9422(03)00300-5 12946402

[B60] Yankner B. Lu T. Loerch P. (2008). The aging brain. *Annu. Rev. Pathol.* 3 41–66. 10.1146/annurev.pathmechdis.2.010506.092044 18039130

[B61] Youl E. Bardy G. Magous R. Cros G. Sejalon F. Virsolvy A. (2010). Quercetin potentiates insulin secretion and protects INS-1 pancreatic β-cells against oxidative damage via the ERK1/2 pathway. *Br. J. Pharmacol.* 161 799–814. 10.1111/j.1476-5381.2010.00910.x 20860660 PMC2992896

[B62] Zafra-Stone S. Yasmin T. Bagchi M. Chatterjee A. Vinson J. Bagchi D. (2007). Berry anthocyanins as novel antioxidants in human health and disease prevention. *Mol. Nutr. Food Res.* 51 675–683. 10.1002/mnfr.200700002 17533652

