# Corrigendum: Mechanosensory Neuron Aging: Differential Trajectories with Lifespan-Extending Alaskan Berry and Fungal Treatments in *Caenorhabditis elegans*

**DOI:** 10.3389/fnagi.2017.00158

**Published:** 2017-05-23

**Authors:** Courtney Scerbak, Elena Vayndorf, Alicia Hernandez, Colin McGill, Barbara Taylor

**Affiliations:** ^1^Institute of Arctic Biology, University of Alaska FairbanksFairbanks, AK, United States; ^2^Department of Biology and Wildlife, University of Alaska FairbanksFairbanks, AK, United States; ^3^Department of Biology, Earlham CollegeRichmond, IN, United States; ^4^Chemistry Department, University of Alaska AnchorageAnchorage, AK, United States

**Keywords:** mechanosensory neuron, *C. elegans* neuron aging, neuron morphology, nutrition, blueberry, lowbush cranberry, chaga, crowberry

In the original article, there was a mistake in Table 2 as published. The N, mean lifespan, percent of control, and *p*-value listed in the first columns of this table are meant to align with the Kaplan-Meier survival curves shown in Figures 1A–C. However, the percentages listed in the published table (for all of the blueberry treatments) do not match up with the mean lifespans listed. The corrected Table 2 appears below. The authors apologize for this error and state that this does not change the scientific conclusions of the article in any way.

**Table 2:** **Alaskan berry and fungus treatments extend wildtype *C. elegans* lifespan**.

**Treatment**	***N***	**Mean lifespan ± S.E.M**.	**Percent of control (%)**	***p*-value**	**Highest increase observed (%)**	**Lowest increase observed (%)**
**Blueberry dose (**μ**g/mL)**						
0	60	9.48 ± 0.31				
60	66	12.4 ± 0.56	130	<0.0001	128	107
100	60	11.2 ± 0.59	118	<0.0001	144	111
200	51	12.4 ± 0.73	130	0.001	147	120
400	58	11.2 ± 0.58	118	<0.0001	139	120
800	60	9.31 ± 0.28	98	0.341	117	109 (n.s.)
**Lowbush cranberry dose (**μ**g/mL)**						
0	50	11.3 ± 0.58				
50	50	13.9 ± 0.91	123	0.012	122	116
100	51	11.1 ± 0.50	98	0.868	116	104 (n.s.)
200	50	12.5 ± 0.49	110	0.123	119	106
400	50	13.9 ± 0.89	123	0.016	122	108
800	50	12.5 ± 0.28	110	0.229	119	108 (n.s)
**Chaga dose (**μ**g/mL)**						
0	50	10.7 ± 0.50				
50	49	13.1 ± 0.60	122	0.002	122	113
200	50	12.9 ± 0.55	121	0.005	124	117
800	47	12.9 ± 0.65	121	0.003	121	116

*Percent change in lifespan relative to control, significance from the representative survival curves shown in Figures 1A–C. The highest and lowest percent increase in lifespan relative to control in all replicates is also reported. Experiments were repeated in multiple independent trials (≥3), all with the same directional effect and similar magnitude of effect. Statistical significance (p-value) calculated with Kaplan-Meier log-rank test*.

## Conflict of interest statement

The authors declare that the research was conducted in the absence of any commercial or financial relationships that could be construed as a potential conflict of interest.

