# Supplementary material for: Mechanosensory Neuron Aging: Differential Trajectories with Lifespan-Extending Alaskan Berry and Fungal Treatments in Caenorhabditis elegans
Source: Front Aging Neurosci. 2016 Jul 18;8:173. doi: 10.3389/fnagi.2016.00173 (PMC4947587; doi:10.3389/fnagi.2016.00173)
Supplement: Supplementary file 1 [file Image_1.PDF]

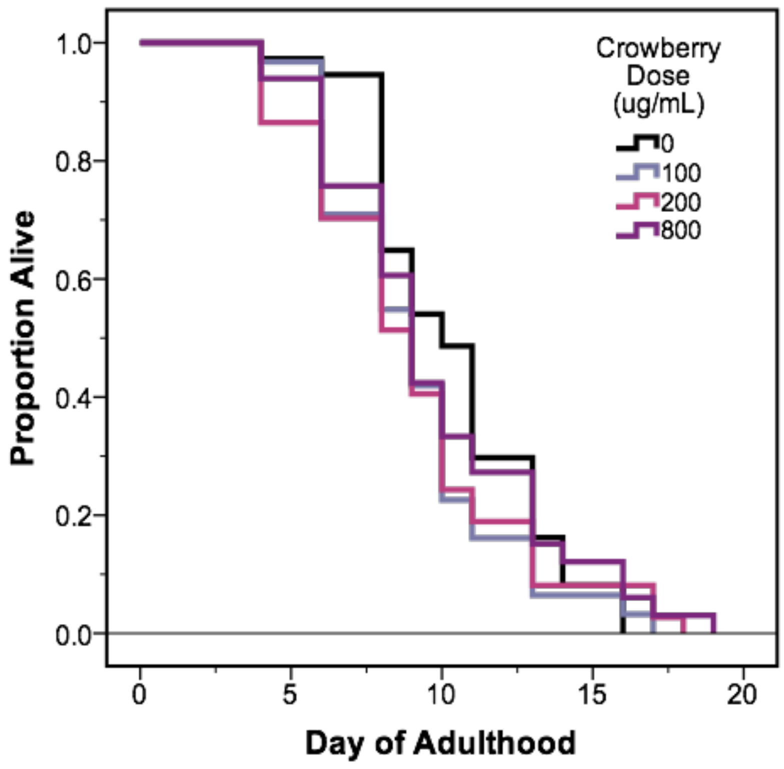

Supplemental Figure 1 Crowberry treatment does not affect wildtype *C. elegans* lifespan. Treatment with 100, 200, and 800μg/mL crowberry mixed into NGM agar did not significantly affect mean or median lifespan at 25°C ($0.13<p<0.78$), Kaplan-Meier log-rank test; 3 replicates). Representative survival curves are shown (N=50 per treatment group).
