# Supplementary material for: Mechanosensory Neuron Aging: Differential Trajectories with Lifespan-Extending Alaskan Berry and Fungal Treatments in Caenorhabditis elegans
Source: Front Aging Neurosci. 2016 Jul 18;8:173. doi: 10.3389/fnagi.2016.00173 (PMC4947587; doi:10.3389/fnagi.2016.00173)
Supplement: Supplementary file 2 [file Image_2.PDF]

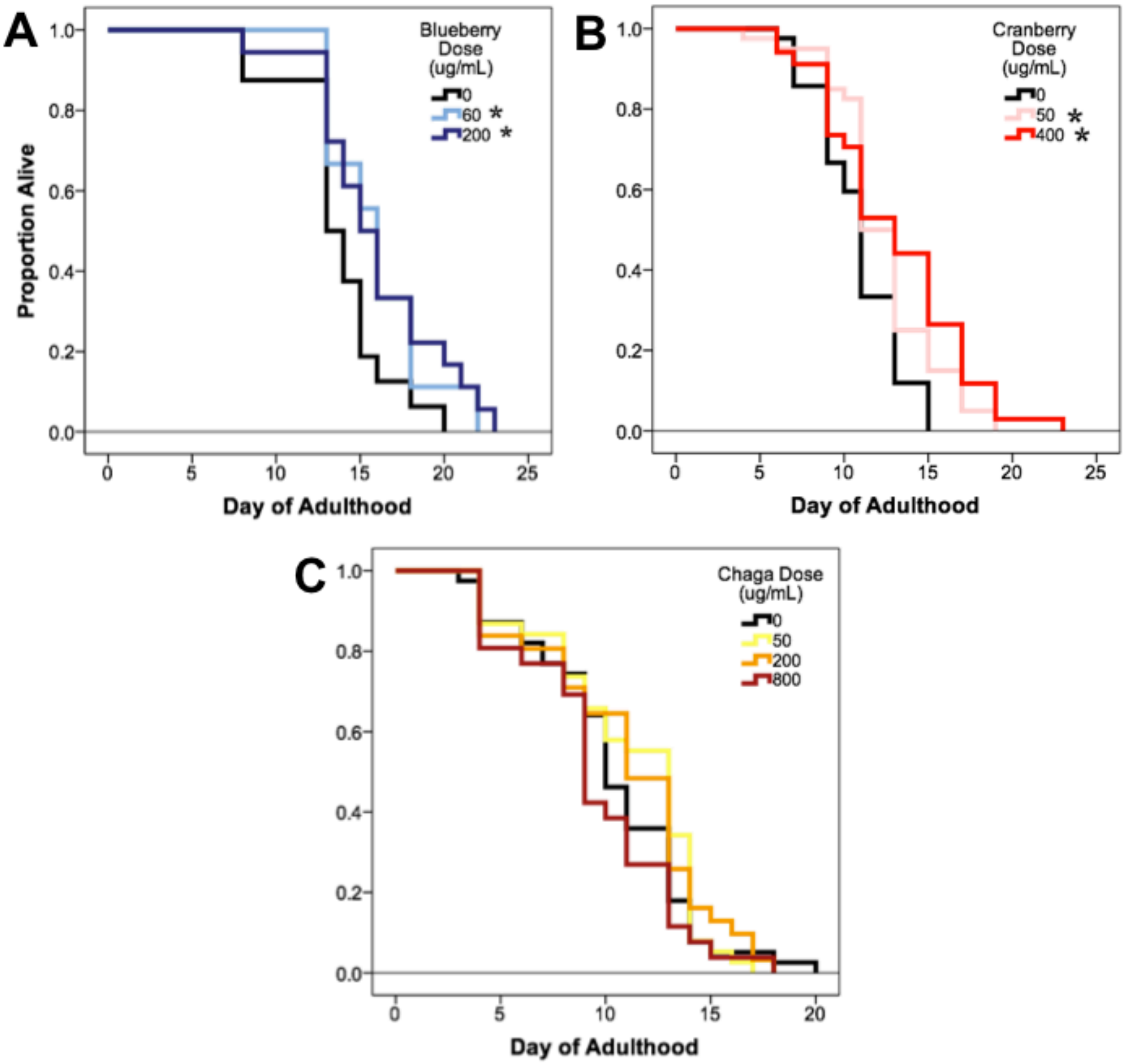

Supplemental Figure 2 Blueberry and lowbush cranberry treatments increase wildtype *C. elegans* lifespan even with UV killed bacterial food source. Mean *C. elegans* lifespan cultured on UV killed bacteria increased from 10.2±0.3 to 11.4±0.6 and 12.3±0.3 days (11.7% and 20.2% increase) upon treatment with 60 and 200µg/mL blueberry, respectively (p<0.05, Kaplan Meier log-rank test). 50 and 400µg/mL lowbush cranberry treatment increased lifespan to 12.6±0.5 and 13.2±0.7 days (14.2% and 19.9% increase), respectively, from 11.1±0.3 days in the presence of UV killed bacteria (p<0.01, Kaplan Meier log-rank test). Treatment with 50, 200, and 800µg/mL chaga did not significantly alter lifespan in the presence of UV killed bacteria (p>0.25, Kaplan Meier log-rank test, 3 replicates). Asterisks represent significance from untreated control (p<0.05, Kaplan Meier log-rank test). Representative survival curves are shown (N=50 for each treatment group).
