# Supplementary material for: Mechanosensory Neuron Aging: Differential Trajectories with Lifespan-Extending Alaskan Berry and Fungal Treatments in Caenorhabditis elegans
Source: Front Aging Neurosci. 2016 Jul 18;8:173. doi: 10.3389/fnagi.2016.00173 (PMC4947587; doi:10.3389/fnagi.2016.00173)
Supplement: Supplementary file 3 [file Image_3.PDF]

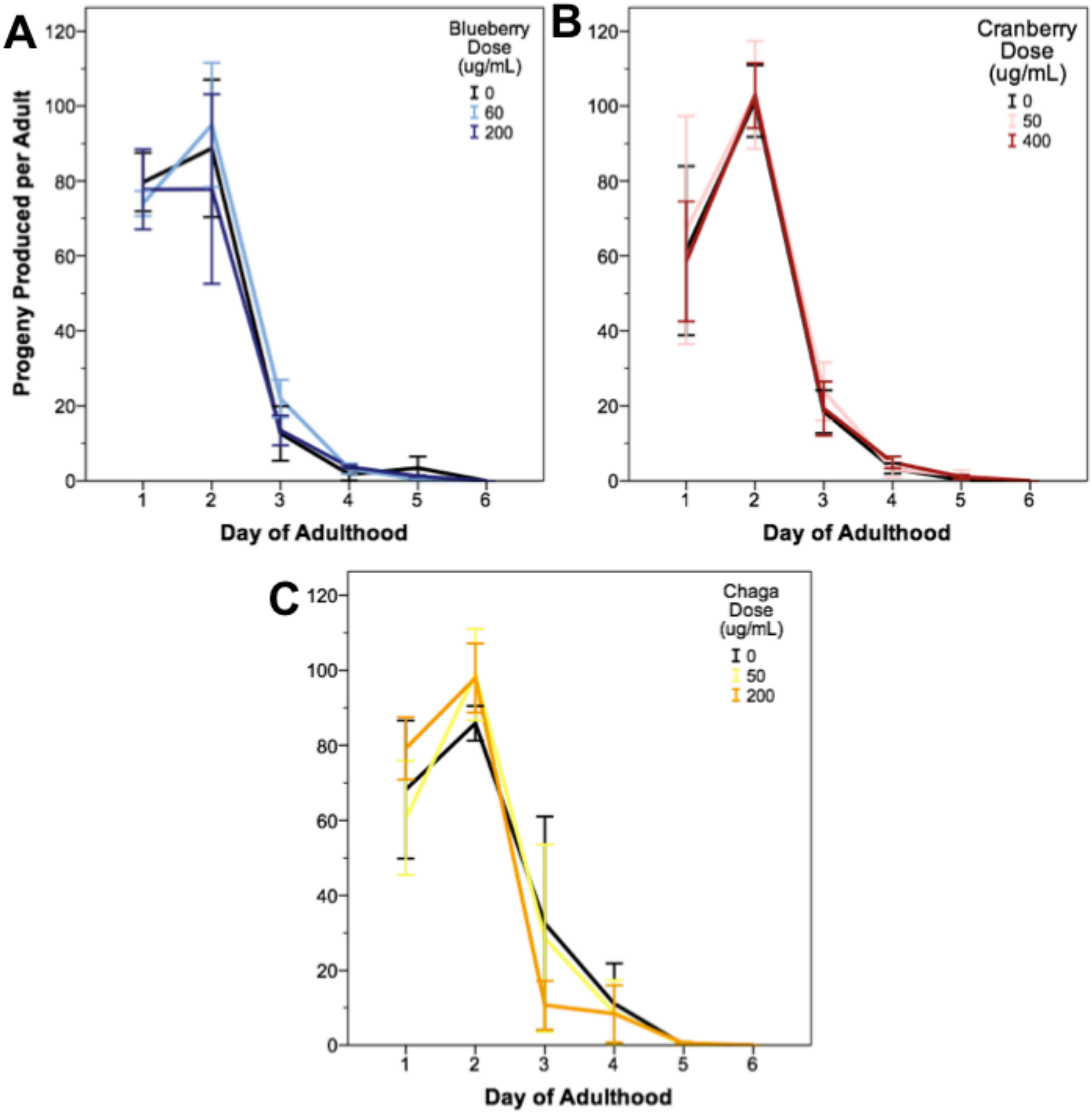

Supplementary Figure 3 Lifespan-extending Alaskan berry and fungus treatments alter wildtype C. elegans daily progeny production, but not total progeny produced. Blueberry (A), lowbush cranberry (B), and chaga (C) treatments differentially affected the number of progeny produced per adult per day on days 2, 3, and 1 of adulthood, respectively ($p<0.05$; Poisson log linear model), but did not alter the total number of progeny produced per adult (Table 3). Peak progeny production in all groups was observed at day 2 of adulthood (48h after treatment administration) and all animals ceased viable progeny production by day 6 of adulthood.
